# Immune-Enhancing Effects of a High Molecular Weight Fraction of *Cynanchum wilfordii* Hemsley in Macrophages and Immunosuppressed Mice

**DOI:** 10.3390/nu8100600

**Published:** 2016-09-27

**Authors:** Mi Jang, Tae-Gyu Lim, Sungeun Ahn, Hee-Do Hong, Young Kyoung Rhee, Kyung-Tack Kim, Eunjung Lee, Jeong Hoon Lee, Yun Ji Lee, Chan Sik Jung, Dae Young Lee, Chang-Won Cho

**Affiliations:** 1 Traditional Food Research Center, Korea Food Research Institute, Seongnam 13539, Gyeonggi, Korea; jangmi@kfri.re.kr (M.J.); tglim83@kfri.re.kr (T.-G.L.); honghd@kfri.re.kr (H.-D.H.); ykrhee@kfri.re.kr (Y.K.R.); tack@kfri.re.kr (K.-T.K.); ejlee@kfri.re.kr (E.L.); 2 Department of Oriental Medicinal Biotechnology, College of Life Sciences, Kyung Hee University, Yongin 17104, Gyeonggi, Korea; se8688@gmail.com; 3 Department of Herbal Crop Research, National Institute of Horticultural and Herbal Science, RDA, Eumseong 27709, Chungbuk, Korea; artemisia@korea.kr (J.H.L.); yoong0625@korea.kr (Y.J.L.); jung100@korea.kr (C.S.J.); 4 Herbal Crop Utilization Research Team, National Institute of Horticultural and Herbal Science, RDA, Eumseong 27709, Chungbuk, Korea; dylee0809@korea.kr

**Keywords:** *Cynanchum wilfordii*, polysaccharide, immunostimulatory activity, cytokine, nitric oxide, cyclophosphamide

## Abstract

The objective of this study was to investigate the immune-enhancing activity of a high molecular weight fraction (HMF) of *Cynanchum wilfordii* in RAW 264.7 macrophages and the cyclophosphamide (CYC)-induced mouse model of immunosuppression. To identify the bioactive substances of HMF, a crude polysaccharide (HMFO) was obtained and treated with sodium periodate (an oxidation agent) or digested with protease. In macrophages, HMF treatment enhanced the production of nitric oxide (NO) and cytokines (tumor necrosis factor alpha (TNF-α), interleukin 6 (IL-6), and interleukin 1β (IL-1β)), as well as phagocytic ability. In CYC-immunosuppressed mice, HMF improved relative spleen and thymus weights, natural killer (NK) cell activity, and splenic lymphocyte proliferation. These increases in NO and cytokines were mediated by up-regulation of nuclear factor kappa B (NF-κB) and mitogen-activated protein kinase (MAPK) signaling pathways. Periodate treatment, but not protease treatment, decreased the immune-enhancing activity of HMFO, suggesting that polysaccharides are the active ingredients in *C. wilfordii* extract.

## 1. Introduction

The immune system is the host’s defense against various infectious organisms, such as bacteria, viruses, parasites, and fungi, and the immune response triggered by these infectious sources can cause disease [1]. The level of the immune response can be adjusted and maintained to a desired level by immunomodulation, which is very important for promoting health. There is a close relationship between the immune system and disease [2]. Cytokines, which are produced by immune cells and have immunomodulatory and anti-inflammatory abilities, play an important role in the immune response and allow for the integration of the behavior of cells in time. Activated macrophages, T lymphocytes, and natural killer (NK) cells mainly produce the cytokines such as tumor necrosis factor alpha (TNF-α), interleukin 6 (IL-6), and IL-1β, and they play an important role in the cellular immune process by aiding the elimination of abnormal cells [3]. Suppression of the immune system induces significant changes in the human body. Immune-suppression has been shown to be caused by various external stresses, including pesticides, alcohol and tobacco abuse, antibiotics, chemotherapy, birth control pills, cortisone, and other drug therapies [4,5,6]. Recently, herbal extracts have been studied extensively for their potential therapeutic effects in immune function. The immunomodulatory effects of traditional herbal medicines, such as echinacea, ginseng, and astragalus, have been investigated as agents against infections and neoplastic diseases [7].

As per the Korean Pharmacopoeia [8], *Cynanchi wilfordii* Radix is the root of *Cynanchum wilfordii* Hemsley. The root of *C. wilfordii* has been used widely as a traditional herbal medicine, and previous studies have demonstrated that it possesses properties as a blood tonic, with vital essence enriching and immune-enhancing activities [9]. In addition, antitumor, anti-oxidative, anti-diabetes mellitus, and anti-inflammatory activities have been attributed to the root of *C. wilfordii* [9,10,11,12,13,14]. The roots of *C. wilfordii* contain several biologically active compounds, such as gagaminine and its glycosides, wilfosides and cynauricuosides, as well as sarcotine, penupogenin, and cynandione A [15]. Previously, it was reported that acetophenones, cynandione A, and its derivatives exhibited neuroprotective and anti-tumor activities [11,16,17]. Although enhancement of the immune response by *C. wilfordii* has been reported, its related immune-modulatory activities in vitro and in vivo remain unclear. In this study, we investigated the immunostimulatory activity of a high-molecular-weight fraction (HMF) of *Cynanchum wilfordii* Radix in RAW 264.7 macrophages and a cyclophosphamide (CYC)-induced immunosuppressed mouse model. For further study, we also explored the immunobioactive substance of high molecular weight fraction HMF, a crude polysaccharide (HMFO) obtained from HMF, and its immunostimulatory effect and underlying molecular mechanisms in RAW 264.7 macrophages.

## 2. Materials and Methods

### 2.1. Chemicals and Reagents

Dulbecco’s Modified Eagle’s Medium (DMEM), fetal bovine serum (FBS), penicillin, and streptomycin were obtained from Life Technologies (Carlsbad, CA, USA). The primary antibodies against inducible nitric oxide synthase (iNOS), total p38, phosphorylated-c-Jun *N*-terminal kinase (JNK), phosphorylated-extracellular signal regulated kinase (ERK)1/2, total JNK, total ERK1/2, and β-actin were purchased from Santa Cruz Biotechnology (Dallas, TX, USA). Antibodies against phosphorylated-inhibitor of kappa B (IκB)-α, phosphorylated-IκB kinase (IKK)-α/β, and phosphorylated-p38 were purchased from Cell Signaling Technology, Inc. (Beverly, MA, USA). Enzyme-linked immunosorbent assay (ELISA) kits for TNF-α and IL-6 were obtained from BD Biosciences (San Jose, CA, USA). An OptEIA mouse ELISA kit for IL-1β was obtained from R&D Systems (Minneapolis, MN, USA).

### 2.2. Preparation of a High Molecular Weight Fraction of Cynanchum wilfordii

The roots of *Cynanchum wilfordii* (CW) were cultivated in Jeongseon, Gangwon Province, Korea, for 2 years, harvested in August 2015, and authenticated by Dr. Jung Hoon Lee, National Institute of Horticultural and Herbal Science (NIHHS), Rural Development Administration (RDA) of Korea. The dried roots of CW (1 kg) were extracted twice with 44 L of water solution at 95 °C for 6 h. After extraction, the solution was filtered and concentrated using a rotary evaporator under vacuum at 55 °C. The filtered concentrate was lyophilized with a freeze dryer at −35 °C, and the yield of the dried powder was approximately 25.1% (*w*/*w*). Commercial ultrafiltration was used to collect and concentrate a high molecular weight fraction. The dried powder of CW extract in distilled water (DW) (20 g/4 L) was centrifuged at 6350× *g* for 5 min and then the supernatant was processed through polyethersulfone ultrafiltration (UF) membranes with a molecular weight cut-off (MWCO) of 30 kDa (Sartocon 3081465902E-SG; Sartorius, Göttingen, Germany) in a cross-flow filtration system. The ultra-filtered retentates were freeze-dried, and the high molecular weight fraction, named HMF, was used for the experiments.

### 2.3. Extraction of a Crude Polysaccharide

A crude polysaccharide (HMFO) was obtained from the HMF of CW. The HMF was precipitated by the addition of four volumes of 95% ethanol. The mixture was allowed to stand at 4 °C overnight and then was centrifuged at 6350× *g* for 20 min to obtain the precipitate. The precipitate was lyophilized to produce HMFO.

### 2.4. Chemical Analyses

Total sugar content was determined by the phenol-sulfuric acid method [18] using glucose as a standard. Uronic acid content was measured by the m-hydroxydiphenyl sulfuric acid method as modified by Taylor and Buchanan-Smith [19] using galacturonic acid as the standard. Protein content was determined with the bicinchoninic acid (BCA) protein assay kit (Pierce Biotechnology, Rockford, IL, USA), using bovine serum albumin (BSA; Sigma-Aldrich, St. Louis, MO, USA) as a standard [20]. The content of 2-keto-3-deoxy-d-manno-2-octulosonic acid (KDO) was determined colorimetrically by the modified thiobarbituric acid (TBA) method [21] using 2-keto-3-deoxyoctonate ammonium salt as a standard.

### 2.5. Monosaccharide Composition and Molecular Weight Distribution

In order to carry out H_2_SO_4_-hydrolysis, the samples were mixed with 72% H_2_SO_4_ at 30 °C for 2 h and then the reaction mixtures with added DW were hydrolyzed at 120 °C for 1 h. The monosaccharide composition was identified and quantified using high-performance anion exchange chromatography coupled with pulsed amperometric detection (HPAEC-PAD). The hydrolysates (25 μL) were applied onto a Dionex ICS-5000 system fitted with a CarboPac PA1 analytical column (4 mm × 250 mm, Dionex Co., Sunnyvale, CA, USA) combined with a Dionex CarboPac PA1 Guard Column (4 mm × 50 mm, Dionex Co.). The monosaccharides were separated isocratically using 18 mM NaOH and a constant flow rate of 1.0 mL/min. A mixture of fucose, rhamnose, arabinose, galactose, glucose, xylose, mannose, and fructose was used as a standard. In addition, separation and quantification of galacturonic acid and glucuronic acid was performed in a Dionex ICS-5000 system as described above, except the mobile phase was 100 mM NaOH and 100 mM NaOAc. Molecular weight distribution was determined using high performance gel permeation chromatography (HPGPC) equipped with a refractive index detector (RID). The sample solution was injected on two serially linked Shodex OHpak SB-803 HQ and SB-805 HQ (8.0 mm × 300 mm, Showa Denko Co., Tokyo, Japan). The injection volume was 20 μL, and the flow rate of 0.1 M sodium chloride eluent was 0.5 mL/min at 30 °C. The MW was calculated by the calibration curve obtained by using a Shodex standard pullulan kit with P-800 (MW 853,000), P-400 (MW 380,000), P-200 (MW 186,000), P-100 (MW 100,000), P-50 (MW 48,000), P-20 (MW 23,700), P-10 (MW 12,200), and P-5 (MW 4,800) (Showa Denko Co.) as standards.

### 2.6. Protease Treatment and Periodate Oxidation of HMFO

For protease treatment, HMFO (50 mg) was dissolved in 10 mM sodium acetate buffer with 5 mM calcium acetate at pH 7.5, and 50 mg of protease (protease type XIV from *Streptomyces griseus*, Sigma-Aldrich) was added. After incubation at 37 °C for 48 h, the digest was heated in a boiling water bath for 5 min to stop the enzymatic reaction. The solution was centrifuged at 400× *g* for 15 min, and the supernatant was lyophilized after dialysis. To conduct periodate oxidation, 50 mg of HMFO was dissolved in 30 mL of 50 mM acetate buffer (pH 4.5) and then 75 mM sodium periodate (10 mL) was added. After the reaction mixture was incubated at 4 °C in the dark for 96 h, 5 mL of ethylene glycol was added to destroy excess periodate and then the mixture was dialyzed against DW for 72 h. Non-dialyzable solution was concentrated to 20 mL, and 20 mg of sodium tetrahydridoborate was added to the concentrate. The reaction mixture was stirred for 12 h at room temperature and then neutralized with acetic acid. The oxidized product was obtained as a lyophilizate after dialysis.

### 2.7. Cell Culture

The RAW 264.7 murine macrophage cells were purchased from the Korean Cell Line Bank (KCLB, Seoul, Korea) and cultured in DMEM medium supplemented with 10% FBS, penicillin (100 units/mL), and streptomycin sulfate (100 µg/mL) at 37 °C in a humidified incubator (5% CO_2_).

### 2.8. Cell Viability Assay

Cell viability was measured using the CCK-8 (Cell Counting Kit, Dojindo, Tokyo, Japan)-based colorimetric assay. RAW 264.7 cells were seeded in a 96-well plate at a density of 1 × 10^4^ cells per well and were treated with various concentrations of HMF or HMFO for 24 h. Cells were incubated with the CCK-8 reagent for 2 h, and optical density (OD) was determined at 450 nm (Infinite M200, Tecan Trading AG, Männedorf, Switzerland). The OD of the samples was compared to that of the untreated control to obtain the percentage viability.

### 2.9. Measurement of Nitric Oxide (NO)

The presence of nitrite, a stable oxidized product of NO, was determined in cell culture media by Griess reagent. RAW 264.7 macrophages (1 × 10^5^ cells/mL) were cultured in 24-well plates and stimulated with HMF, HMFO, protease-treated HMFO, or periodate-oxidized HMFO for 24 h. One hundred microliters of culture supernatant were collected and mixed with an equal volume of Griess reagent (0.1% *N*-(1-naphthyl) ethylenediamine dihydrochloride, 1% sulfanilamide, and 2.5% H_3_PO_4_). After incubation for 15 min at room temperature, the OD was measured at 540 nm using a microplate reader. Nitrite concentrations in the supernatants were determined by comparison with a sodium nitrite standard curve.

### 2.10. Determination of TNF-α, (IL-6) and IL-1β Production

RAW 264.7 macrophages (1 × 10^5^ cells/mL) were cultured in 24-well plates and stimulated with HMF, HMFO, protease-treated HMFO, or periodate-oxidized HMFO. Supernatants were collected after 24 h, and the levels of TNF-α, IL-6 and IL-1β were measured by ELISA kits (R&D Systems, Minneapolis, MN, USA) according to the manufacture’s protocols.

### 2.11. Phagocytosis Assay in RAW 264.7 Cells

The phagocytic ability of RAW 264.7 macrophages was detected using the CytoSelect™ 96-well phagocytosis assay kit (Cell Biolabs Inc., San Diego, CA, USA), following the manufacturer’s instructions. RAW 264.7 macrophages (1 × 10^4^ cell/well) were plated in 96-well plates and incubated overnight at 37 °C to allow adherence to the plate. The cells were pre-incubated with a series of concentrations of HMF (final concentrations of 50, 100, and 200 μg/mL) as well as a negative control (complete DMEM) and positive control (lipopolysaccharide (LPS), 1 μg/mL) for 24 h. Subsequently, non-opsonized zymosan particles were added incubated at 37 °C for 2 h. The amount of engulfed zymosan particles was determined using a colorimetric assay at an absorbance of 405 nm.

### 2.12. Western Blot Analysis

The cells were incubated with HMFO (50, 100, and 200 μg/mL) for 24 h, collected by centrifugation, and washed once with phosphate buffered saline (PBS). Washed cell pellets were lysed for 30 min on ice with Pro-Prep™ protein extraction solution according to the protocol provided. Cell lysates were centrifuged at 15,700× *g* at 4 °C for 5–10 min, and clear supernatants were collected in new tubes and stored at −70 °C. For immunoblotting, proteins in cell lysates were resolved by sodium dodecyl sulfate (SDS)-polyacrylamide gel electrophoresis (PAGE) on 8%–10% gels and then transferred to nitrocellulose membranes (Millipore, Billerica, MA, USA) for 1.5 h. Immunoblots were incubated for 1 h with blocking solution (5% skim milk) at room temperature and then incubated overnight with a 1:1000 dilution of primary antibody at 4 °C. Blots were washed three times with Tween 20/Tris-buffered saline (T/TBS) and then incubated with a 1:2000 dilution of horseradish peroxidase (HRP)-conjugated secondary antibody (Santa Cruz Biotechnology Inc.) for 90 min at room temperature. Finally, blots were visualized using an enhanced chemiluminescence system (Amersham Biosciences Inc., Piscataway, NJ, USA) followed by exposure to X-ray film (Fuji Photo Film Co., Ltd., Tokyo, Japan).

### 2.13. Animals and Treatments

Female BALB/c mice (8 weeks old, 18–20 g) were purchased from Koatech Animal Inc. (Pyeongtaek, Korea). The mice were housed at 22 ± 1 °C, with 12 h-light/12-h dark cycle, 50%–60% relative humidity, and given free access to food and water during the experiments. All animal studies were performed in accordance with the Guiding Principles for the Care and Use of Laboratory Animals of the Ethics Committee of the Korea Food Research Institute.

Animal experiments were conducted using immunosuppressed mice. Mice were randomly divided into five groups (eight mice in each group). Normal control mice did not receive any treatment for immunosuppression. The remaining four groups were injected intraperitoneally with 150 mg/kg body weight CYC, a known immunosuppressant, on days 8, 9, and 10 after administration of the respective treatment. The immunosuppressed control group mice received normal saline for a period of 28 days. The immunosuppressed model positive control mice were orally administered 200 mg/kg body weight/day CVT-E002™ for 28 days. CVT-E002™ is an immunostimulatory polysaccharide-rich extract of the root of North American ginseng (*Panax quinquefolius*) [22]. The two HMF treatment groups were orally administered 100 or 200 mg/kg body weight/day HMF for 28 days. At the end of the study, mice were weighed and sacrificed by cervical dislocation. Relative thymus and spleen weights were calculated according to the following formula: Relative (%) = (thymus or spleen weights (g)/body weight (g)) × 100. The collected spleen samples were used to measure the splenocyte proliferation and NK cell activity.

### 2.14. Preparation of Mouse Splenocytes

The spleen was gently homogenized, and the cell suspension was passed through a 100-μm nylon cell strainer (BD Falcon, San Jose, CA, USA) to obtain single cell suspensions. After centrifugation, the red blood cells were lysed in Red Cell Lysis Buffer (Hybri-Max™, Sigma-Aldrich). After washing twice with serum-free Roswell Park Memorial Institute (RPMI) 1640 medium, the splenocytes were harvested and resuspended in RPMI 1640 medium (with 10% FBS, 100 unit/mL penicillin, and 100 μg/mL streptomycin).

### 2.15. Splenocyte Proliferation Assay

Splenocyte proliferation was assessed using the CCK-8 kit (Dojindo Laboratories, Tokyo, Japan) according to manufacturer’s instructions. The cell number was briefly adjusted to a density of 3 × 10^6^ cells/mL of medium, and 1 mL of spleen cell suspension was seeded in 24-well plates and cultured with 75 μL of the mitogen solutions or sterile DW for control culture. The mitogens were concanavalin A (Con A) and LPS, which were dissolved in sterile DW to a concentration of 100 μg/mL and 400 μg/mL, respectively. Con A and LPS were used for measuring the proliferation of T and B lymphocytes in splenocyte cultures, respectively. After incubation at 37 °C in a humidified incubator with 5% CO_2_ for 72 h, aliquots (100 μL) of CCK-8 reagent were added to the cells and then cultured in the incubator for 4 h. The absorbance at 450 nm was measured on a microplate reader.

### 2.16. Flow Cytometric Analysis

Flow cytometry-assisted analysis was performed using 1 × 10^6^ splenocytes suspended in 100 μL of staining buffer (0.5% BSA, 0.04% EDTA, 0.05% sodium azide in PBS), which was pre-incubated with anti-mouse Fc Block FcR blocking reagent (BD PharMingen, San Diego, CA, USA) for 20 min. Surface stains included FITC-conjugated anti-mouse CD3 (eBioscience, San Diego, CA, USA), PerCP-Cy™ 5.5-conjugated anti-mouse CD8 (BD PharMingen), and APC-conjugated anti-mouse CD4 antibodies (BD PharMingen). The cells were then fixed and permeabilized with BD Cytofix/Cytoperm solution (BD Biosciences) for 20 min at 4 °C and washed once. Stained cells were analyzed by flow cytometry using a FACSCanto II flow cytometer (Becton Dickinson, Mountain View, CA, USA). The acquired data were analyzed with CellQuest PRO software. For analysis of T cells, cells were gated to be CD3+, and this population was analyzed for expression of CD4 and CD8. This is particularly true for antibodies to CD3, a marker commonly used to identify T cell receptor-bearing cells. The results were expressed as a percentage of CD3 + CD4 + or CD3 + CD8 + cells.

### 2.17. NK Cell Activity

YAC-1 lymphoma cells (KCLB NO. 40160) were purchased from the Korean Cell Line Bank. The cell lines were grown in RPMI 1640 medium with 10% FBS and 1% penicillin-streptomycin and incubated at 37 °C in 5% CO_2_. YAC-1 cells were used as the target cells, while splenocytes were the effector cells. Two different ratios of effector to target cell were used. The NK cell activity of the splenocytes was determined by cytotoxicity against NK cell-sensitive YAC-1 cells using a lactate dehydrogenase (LDH) leakage assay [23]. The amount of LDH in the cultured medium was an indicator of cytotoxicity. After the end point of the experiment, the LDH activity of the cells in the culture medium was determined, following the instructions of the LDH cell cytotoxicity assay kit (DoGen, Seoul, Korea). The NK activity of effector cells was calculated by the following formula: cytotoxicity (%) = 100 × {(A − B) − (C − D)}/(E − F); where A is the experimental release, B the spontaneous release of effector cells, C the spontaneous release of target cells, D the blank for the spontaneous release of effector cells, E the maximum release of target, and F is the blank for the maximum release.

### 2.18. Statistical Analysis

All data are presented as the mean ± standard deviation (SD). Data were analyzed using one-way analysis of variance (ANOVA) followed by Scheffe’s test to detect intergroup differences using SPSS software version 20.0 (SPSS Inc., Armonk, NY, USA); *p* < 0.05 was considered statistically significant.

## 3. Results

### 3.1. Effects of HMF on NO Production and Cytokine and Phagocytic Activity

**Figure 1:**
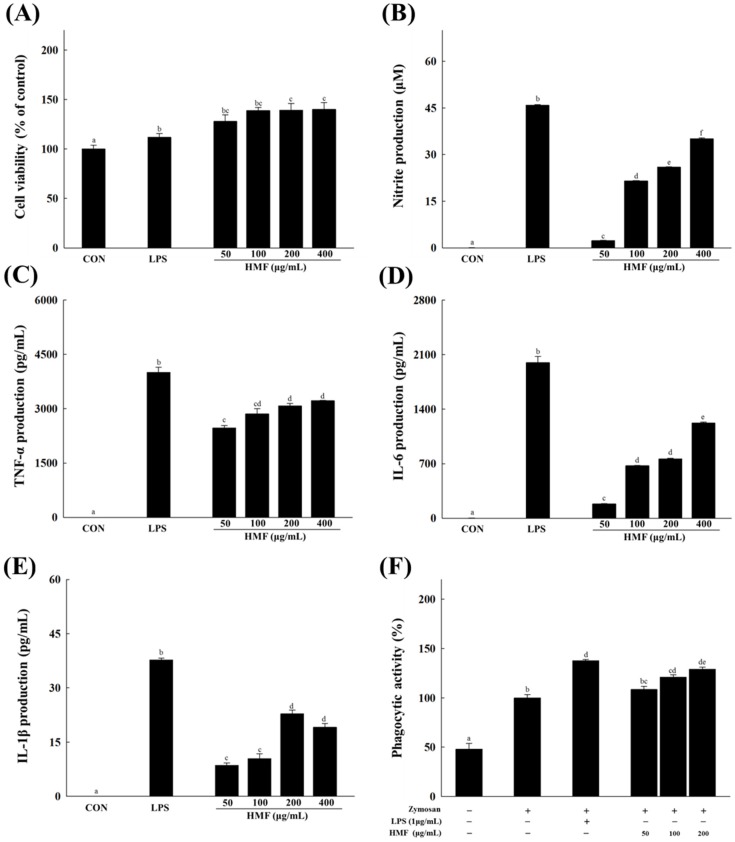
Effects of a high molecular weight fraction (HMF) of *Cynanchum wilfordii* on nitric oxide (NO), tumor necrosis factor alpha (TNF-α), interleukin (IL)-6, and IL-1β production, and phagocytic activity in macrophages. RAW 264.7 macrophages were stimulated with the HMF (50, 100, 200, or 400 μg/mL) or with lipopolysaccharide (LPS; 1 μg/mL). LPS was used as the positive control. (**A**) Cell viability was determined by cell counting kit (CCK)-8 assay; (**B**) NO production was determined by measuring nitrite accumulation in culture medium. The productions of (**C**) TNF-α; (**D**) IL-6; and (**E**) IL-1β were measured using enzyme-linked immunosorbent assays (ELISAs); (**F**) Phagocytic activity was determined using a phagocytosis assay. Values with the different letters are significantly different (*p* < 0.05).

To confirm the immunostimulatory activity of HMF, NO production was evaluated after RAW 264.7 macrophages were treated with HMF. HMF treatment (50, 100, 200 or 400 μg/mL) dose-dependently increased NO concentration compared to untreated cells (Figure 1B). HMF (up to 400 μg/mL) did not affect cell viability of RAW 264.7 macrophages (Figure 1A), showing that increases in NO production were not due to any cytotoxic effect of HMF. To assess the effect of HMF on immune stimulation, we measured the production of these cytokines in RAW 264.7 macrophages. As seen in Figure 1C–E, treatment of HMF increased the production of all immunostimulatory cytokines (TNF-α, IL-6, and IL-1β) examined compared to untreated cells. As shown in Figure 1F, HMF treatment for 24 h enhanced the phagocytic activity of RAW 264.7 cells. Phagocytosis in macrophages treated with HMF (200 μg/mL) was 1.3-fold higher than that of zymosan-only treated control cells.

### 3.2. Effects of HMF on Decreased Immune Function in CYC-Treated Mice

To confirm the immune-enhancing activity of HMF under physiological conditions, an in vivo study was performed. HMF was orally administered to CYC-immunosuppressed mice, and the effects on average body weight and the relative weights of thymus and spleen were determined (Table 1). CYC-treated mice exhibited a significant reduction in relative spleen and thymus weights compared with the normal group mice. HMF treatment at 100 or 200 mg/kg body weight/day, however, markedly restored relative spleen and thymus weights to normal levels and to levels even higher than the CVT-treated group, the positive control.

**Figure 2:**
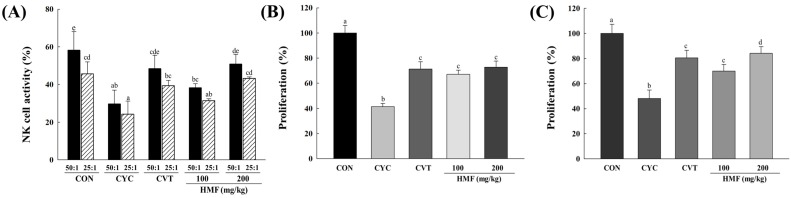
Effects of HMF on natural killer (NK) cell activity and splenic lymphocyte proliferation in CYC-induced mice. HMF (100 mg/kg or 200 mg/kg) or CVT (200 mg/kg) was administered orally once daily for 28 days to CYC-induced mice. (**A**) NK cell activity was determined by lactate dehydrogenase (LDH) assay as described in Materials and Methods. The E:T ratio indicates ratio of effector cells (splenocytes) and target cells (YAC-1 cells); (**B**) Concanavalin A (Con A)-induced T-lymphocyte proliferation; (**C**) LPS-induced B-lymphocyte proliferation. Cell proliferation was measured by using CCK-8 kit. Values with the different letters are significantly different (*p* < 0.05). CYC, cyclophosphamide; CVT, immunostimulatory polysaccharide-rich extract of the root of North American ginseng (*Panax quinquefolius*); HMF, high-molecular-weight fraction of *Cynanchum wilfordii*.

At all effector-to-target (E:T) ratios, treatment with CYC significantly decreased the activity of splenic NK cells compared with mice in the normal control group. However, a dose of 200 mg/kg HMF restored NK cell activity to normal control levels in CYC-treated mice (Figure 2A). As shown in Figure 2B,C, proliferation of T and B lymphocytes in the CYC-treated group was significantly decreased compared with the normal control group. Treatment with 100 and 200 mg/kg HMF enhanced proliferation of T and B lymphocytes significantly compared with the CYC-treated group. Con A and LPS were used to stimulate T and B lymphocyte proliferation, respectively.

**Figure 3:**
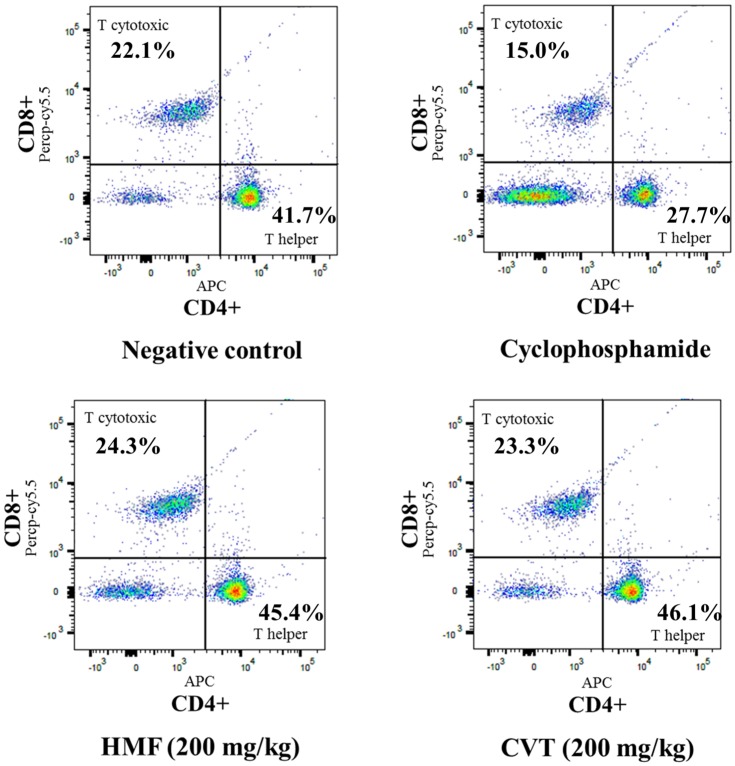
Flow cytometric analyses of spleen CD4+ and CD8+ T cell subsets. Groups of cyclophosphamide, HMF, and CVT mice were immunosuppressed as indicated in the Materials and Methods section. Spleen cells were gated to be CD3+ for analysis of T cells, and this population was analyzed for expression of CD4 and CD8 by flow cytometry. Results were expressed as percentages of CD4+ and CD8+ T cell subsets.

To identify phenotypically distinct subsets of CD4 and CD8 T cells, we carried out flow cytometry analysis. The percentage of helper (CD3+CD4+) T cells was decreased in CYC-treated mice compared to mice in the normal control group: the helper T cell population was 41.7% in normal controls versus 27.7% in CYC-treated group (Figure 3). Treatment with HMF increased the percentage of helper T cells compared with mice in the CYC-treated group. In addition, the percentage of cytotoxic (CD3+CD8+) T cells in the CYC-treated mice was decreased compared with the normal control group, while HMF-treated mice had a higher percentage of cytotoxic T cells than CYC-treated mice.

### 3.3. Effects of a Crude Polysaccharide of HMF on the Immunostimulatory Activity in RAW 264.7 Macrophages

We prepared a crude polysaccharide (HMFO) from HMF by ethanol precipitation and investigated its immunostimulatory effect and underlying molecular mechanisms in RAW 264.7 macrophages.

#### 3.3.1. Effects of Chemical and Enzymatic Treatments of HMFO on Immunostimulatory Activity in RAW 264.7 Macrophages

**Figure 4:**
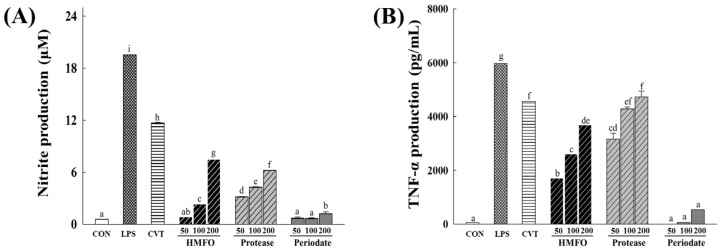

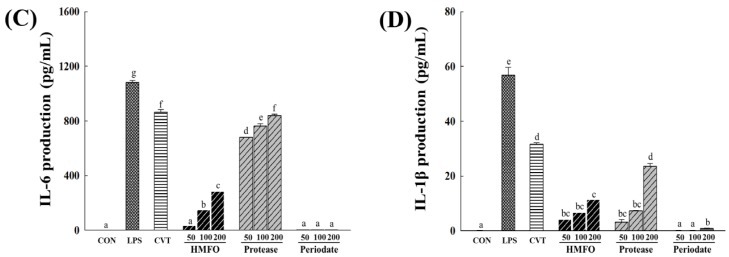
Effects of chemical and enzymatic treatments of HMFO on immunostimulatory activity in RAW 264.7 macrophages. RAW 264.7 macrophages were stimulated with HMFO, protease-treated HMFO, periodate-oxidized HMFO (50, 100, or 200 μg/mL), CVT (200 μg/mL), or LPS (1 μg/mL) for 24 h. The production of NO (**A**) was determined by measuring nitrite accumulation in culture medium. Amounts of TNF-α (**B**); IL-6 (**C**); and IL-1β (**D**) released into culture media were determined by ELISA. Values with the different letters are significantly different (*p* < 0.05). CVT, immunostimulatory polysaccharide-rich extract of the root of North American ginseng (*Panax quinquefolius*); HMFO, a crude polysaccharide of high molecular weight fraction; Protease, protease treated-HMFO; Periodate, periodate-oxidized HMFO.

To identify the immunobioactive substance of HMFO, HMFO was treated with sodium periodate to degrade the carbohydrate units and digested with protease to hydrolyze proteins. Protease-treated HMFO significantly increased the production of NO and cytokines (TNF-α, IL-6, and IL-1β), but periodate-oxidized HMFO did not affect the levels of NO and all measured cytokines (Figure 4A–D). Periodate oxidation of HMFO decreased the immunostimulatory activities in RAW 264.7 macrophages of HMFO by oxidative depolymerization of polysaccharides. Protease-treated HMFO enhanced immunologic macrophage activation, likely because of the existence of structural moieties required for expression of stimulating activity on macrophages.

#### 3.3.2. Effects of HMFO on NO Production and iNOS Expression in RAW 264.7 Macrophages

**Figure 5:**
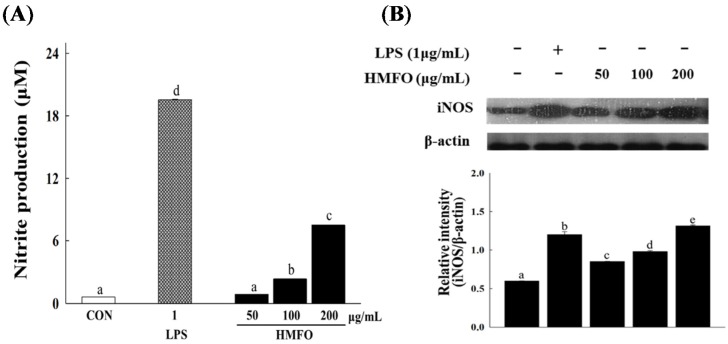

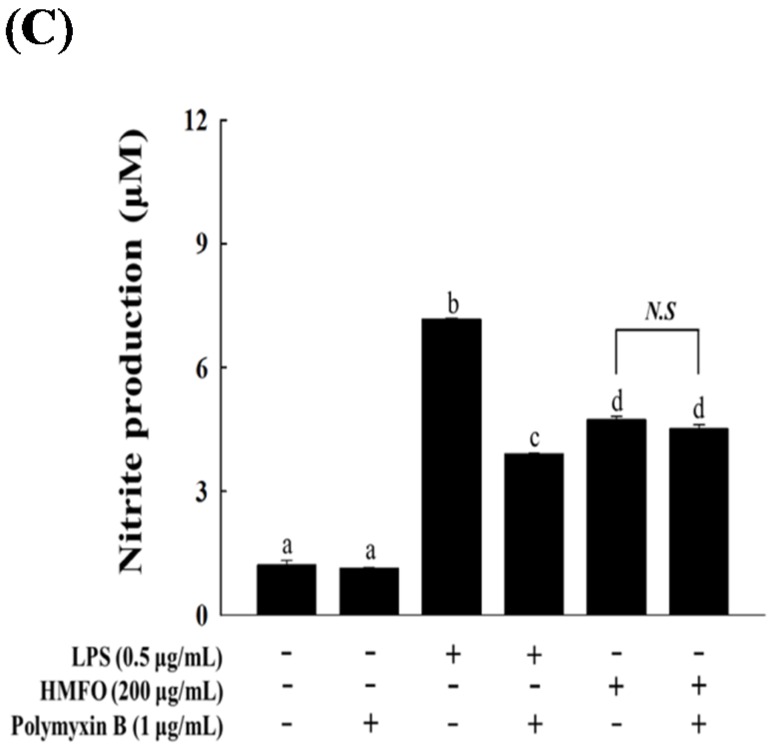
Effects of HMFO on NO production and iNOS expression in RAW 264.7 macrophages. RAW 264.7 macrophages were stimulated with HMFO (50, 100, or 200 μg/mL) and LPS (1 μg/mL) for 24 h. The production of NO (**A**) was determined by measuring nitrite accumulation in culture medium; (**B**) Total proteins were isolated from cells stimulated with HMFO (50, 100, or 200 μg/mL) or LPS (1 μg/mL) for 24 h. β-Actin was used as an internal loading control; (**C**) Cells were pretreated with polymyxin B (1 μg/mL), followed by stimulation with HMFO (200 μg/mL) or LPS (0.5 μg/mL) for 24 h. NO production was determined by measuring nitrite accumulation in culture medium. Values with the different letters are significantly different (*p* < 0.05). HMFO, polysaccharides of high-molecular-weight fraction.

HMFO significantly and concentration-dependently increased the level of NO compared to control (Figure 5A). To determine if this increase in NO secretion was related to modulation of iNOS expression, we measured the protein levels of iNOS in the presence of HMFO (50, 100, or 200 μg/mL) by western blotting. Although iNOS protein was not detectable in unstimulated RAW 264.7 macrophages, HMFO treatment significantly and dose-dependently increased iNOS protein expression (Figure 5B). In addition, HMFO (up to 400 μg/mL) did not affect cell viability of RAW 264.7 macrophages (data not shown), showing that increases in NO production via iNOS expression were not due to any cytotoxic effect of HMFO. Polymyxin B (an LPS inhibitor) markedly inhibited NO production induced by LPS. However, it had no effect on HMFO-induced NO production (Figure 5C). These results indicate that enhanced NO production by HMFO was not the result of endotoxin contamination.

#### 3.3.3. Effects of HMFO on the Phosphorylation of IκB-α and IKK-α/β in RAW 264.7 Macrophages

**Figure 6:**
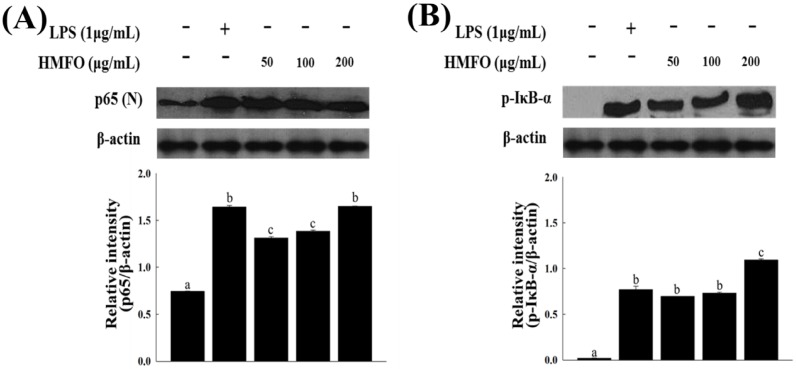

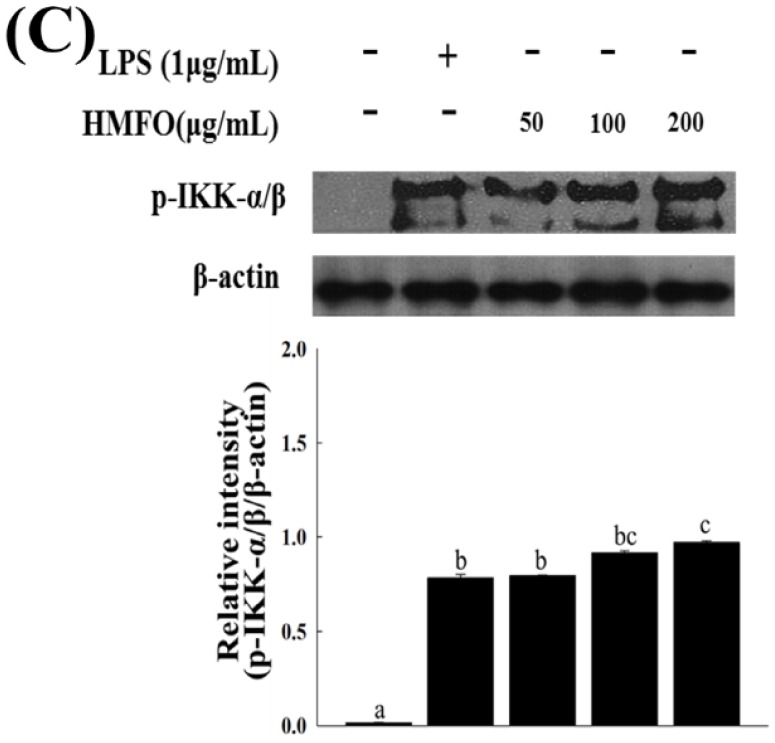
HMFO-induced nuclear factor kappa B (NF-κB) activation by the phosphorylation of inhibitor of kappa B (IκB)-α and IκB kinase (IKK)-α/β in RAW 264.7 macrophages. Cells were stimulated with HMFO (50, 100, or 200 μg/mL) or LPS (1 μg/mL). (**A**) Nuclear extracts (N) were isolated from cells stimulated with HMFO or LPS. Level of p65 in the nuclear fractions was determined by immunoblotting analysis; (**B**,**C**) Phosphorylated IκB-α and IKKα/β isolated from cell lysates were determined by immunoblotting analysis. β-actin was used as an internal control. Values with the different letters are significantly different (*p* < 0.05).

We investigated whether HMFO enhanced nuclear localization of NF-κB and the phosphorylation of IκB-α and IKKα/β in RAW 264.7 macrophages by western blotting. HMFO treatment significantly elevated nuclear translocation of NF-κB and the levels of phosphorylated IκB-α and IKKα/β (Figure 6). Our findings indicate that HMFO regulates NF-κB activation by increasing the nuclear translocation of p65 via IKK-α/β-dependent IκB phosphorylation.

#### 3.3.4. Effects of HMFO on Mitogen-Activated Protein Kinase (MAPK) Phosphorylation in RAW 264.7 Macrophages

**Figure 7:**
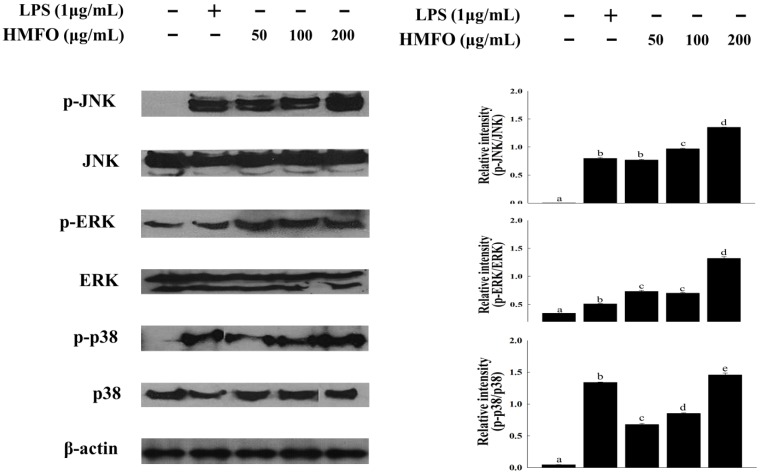
HMFO induced mitogen-activated protein kinase (MAPK) phosphorylation in RAW 264.7 macrophages. Cells were stimulated with HMFO (50, 100, or 200 μg/mL) or LPS (1 μg/mL). Phosphorylated-c-June *N*-terminal kinase (JNK), phosphorylated extracellular signal regulated kinase (ERK), phosphorylated p38, and β-actin isolated from cell lysates were determined by immunoblotting analysis. Values with the different letters are significantly different (*p* < 0.05).

To determine whether HMFO activates the production of NO and cytokines (TNF α, IL-6, and IL-1β) through MAPKs signaling pathways, RAW 264.7 macrophages were cultured as described above, and phosphorylated MAPKs were analyzed using western blot analysis. As shown in Figure 7, HMFO concentration-dependently induced the phosphorylation of ERK, JNK, and p38.

### 3.4. Chemical Compositions and Molecular Weight Distribution

The percentages of neutral sugar, uronic acid, KDO, protein content, and the component sugars of HMFO are summarized in Table 2. More specifically, the monosaccharide composition of HMFO was investigated by HPAEC-PAD. The main monosaccharide components of HMFO were glucose, arabinose, galactose, rhamnose, and galacturonic acid. For HMFO, there were several predominant monosaccharides that contained component sugars characteristic of pectic substances, such as arabinose, galactose, rhamnose, and galacturonic acid. The molecular weight range of HMFO was estimated to be between 11.8 and 520.4 kDa based on the calibration with pullulan standards (Figure S1).

## 4. Discussion

In this study, we demonstrated the immunostimulatory effects of HMF and HMFO on RAW 264.7 macrophages as well as its enhancement of immune function in CYC-immunosuppressed mice.

Activated macrophages secrete various immune mediators, such as NO, TNF-α, IL-1β, and IL-6 [24]. Our results showed that HMF significantly increased the production of NO and immunostimulatory cytokines (TNF-α, IL-1β, and IL-6) in RAW 264.7 macrophages. We also found that HMF enhanced phagocytic activity, which is the initial step of macrophage response to invading antigens. NO production has been linked with many biological functions, including vasodilatation, neurotransmission, immune response, and platelet aggregation [25]. Activated macrophages, T lymphocytes, and NK cells mainly produce TNF-α, IL-6, and IL-1β, and these cytokines play an important role in the cellular immune process by aiding in the elimination of abnormal cells [3]. Macrophages are known to be the most important phagocytes, and phagocytosis by macrophages represents the first and imperative step in the immune response [26]. Macrophages protect the host from infectious agents by phagocytosis, present antigens to lymphocytes, and release numerous cell factors that regulate the activity of other cells [27]. Taken together, these results may explain the immunostimulatory activities of the HMF on macrophages.

To evaluate the immunostimulatory activity of the HMF on a weakened immune system, we used CYC-treated mice, a model of immunosuppression. CYC is a DNA alkylating agent widely used as an anticancer and immunosuppressant, and the thymus and spleen are the two major lymphoid organs severely affected during CYC-induced immunosuppression [28]. The thymus and spleen are important immune organs, and their relative weights are recognized as critical and intuitive indices for nonspecific immunity [29] because immunostimulators have been shown to increase these measures [30]. In this investigation, the relative spleen and thymus weights in the HMF-treated groups were significantly increased compared to those of the CYC-treated group. These results support the conclusion that HMF stimulates the immune system. Elimination of tumor cells is partially mediated by the cytotoxic activity of NK cells and cytotoxic T lymphocytes. One of the main functions of NK cells is to distinguish normal cells from abnormal cells, such as tumor cells, infected cells, and cells that have undergone physical or chemical injury [31,32]. Mouse and human NK cells have been shown in vitro to kill a wide range of tumor cells of hematopoietic and non-hematopoietic origins. In addition, mouse NK cells can remove numerous transplantable and spontaneous tumors in vivo [33,34]. The proliferation of spleen cells is one of the most important steps in the activation pathway of cell-mediated or humoral immunity [35], and the ability of splenic cells to proliferate has been widely used as a method to screen for new immunostimulators, as cell division and DNA synthesis can be stimulated by various antigens, mitogens, and cytokines [36]. Here, oral administration of HMF compensated the decrease in NK cell activity and T- and B-lymphocyte proliferation induced by CYC treatment, which suggests a role for HMF in the activation of NK cell and lymphocytes. CD4+ and CD8+ T cells are the main effectors of the adaptive cellular immune responses. T cell populations have been characterized mainly on the basis of the expression of CD4 or CD8 glycoprotein, which distinguishes helper and cytotoxic/suppressor populations, respectively [37,38]. In this study, treatment with HMF increased the percentage of helper (CD3+CD4+) T and cytotoxic (CD3+CD8+) T cells compared with CYC-treated mice group. These results show that HMF may trigger T cell responses and enhance CD4+ and CD8+ T cell-mediated immunity. Given these data, we speculate that the HMF treatment may compensate CYC-induced immunosuppression and increase immune activities.

In this study, we have found that HMF enhanced the production of NO and cytokines and the phagocytic ability of macrophages and improved relative spleen and thymus weights, NK cell activity, and splenic lymphocyte proliferation in CYC-induced immunosuppressed mice. Among many bioactive substances, polysaccharides in particular have been known to interact with cells of the immune system. These interactions are mainly stimulatory and may potentially act by strengthening innate and adaptive immune responses via direct interaction or by inducing effects via complex reaction cascades [39,40,41]. To confirm whether polysaccharides are responsible for the immune enhancing activities of HMF, we isolated the polysaccharide fraction (HMFO) from HMF and performed periodate oxidation and protease digestion tests. Periodates are powerful oxidizing agents, and periodate oxidation is capable of degrading carbohydrate moieties. Oxidation of carbohydrates by periodate ions is a well-established and routinely used method for structure determination of complex carbohydrates [42]. Proteases degrade proteins by hydrolyzing peptide bonds. In general, exogenously added protease is able to digest proteins exposed on the outer surface of such structures but is prevented from gaining access to proteins on the other side of the membrane barrier. Thus, protease treatment has been used for a variety of purposes, including localizing membrane proteins and enzymes, determining the orientation of transbilayer polypeptides [43]. Given that only periodate-oxidized HMFO, not protease-digested HMFO, decreased the production of NO and immunostimulatory cytokines (TNF-α, IL-6, and IL-1β), polysaccharides may be some of the active ingredients in the aqueous extracts of CW.

To identify the mechanisms involved in the activation of NF-κB by HMFO, we examined its effects on NF-κB activation signals. We found that the activation of NF-κB by HMFO resulted from enhanced IκBα and IKK-α/β phosphorylation and subsequent translocation of p65 to nucleus. NF-κB is a major transcription factor that regulates genes responsible for both the innate and adaptive immune response. In un-stimulated conditions, it is sequestered in the cytoplasm by its inhibitory IκB proteins. However, with stimulation, IκB proteins were phosphorylated by the IKK complex, leading to ubiquitin-dependent IκB degradation by the 26S proteasome [44]. The activation of the NF-κB is dependent on the induction of IκB by IKK, which is a protein complex composed of catalytic subunits IKKα, IKKβ, and a regulatory subunit named NF-κB essential modulator (NEMO) or IKKγ [45]. In this study, HMFO induced the phosphorylation of IKK-α/β, suggesting that HMFO can enhance NF-κB activation by up-regulating IKK-α/β phosphorylation in RAW 264.7 macrophages.

MAPKs are protein Ser/Thr kinases that convert extracellular stimuli into a wide range of cellular responses, and the production of macrophage-related cytokines and chemokines is highly regulated by numerous signaling molecules such as MAPKs, ERK, JNK, and p38 as well as NF-κB [46,47]. When we examined the effects of HMFO on the phosphorylation of MAPK in RAW 264.7 macrophages, we found that HMFO increased the phosphorylation of p38, JNK, and ERK. This result indicates that HMFO exerts its immunostimulatory activities by activating MAPK in RAW 264.7 macrophages.

Pectin substances are a major polysaccharide in plants, and soluble pectins can be easily acquired by hot water extraction. Pectins are known to be made of only high molecular weight compounds (α-d-1,4-polygalacturonic acid), wherein d-galacturonic acids are connected by α-1,4 bonds [48]. However, the pectin present in nature has various oligo- and polysaccharide (rhamnose, galactose, arabinose, etc.) branches covalently bound to straight-chain homogalacturonans [49]. In our study, components of the pectic polysaccharides comprised many parts of HMFO. Given that pectins have various pharmacological properties, including immunostimulatory, anti-metastatic, anti-ulcer, anti-nephrosis activities, and cholesterol-reducing effects [50], the biological activity of HMFO seems likely to stem from pectin substances.

## 5. Conclusions

HMF enhanced the phagocytic capacity of macrophages and increased levels of cytokines and NO. In CYC-induced immunosuppressed mice, HMF restored the impairments in thymus and spleen indices, splenic lymphocyte proliferation, and NK cell activity. More importantly, a crude polysaccharide of HMF contributed to its immune-enhancing effect by stimulating macrophages through up-regulation of the NF-κB or MAPK signaling pathways. These findings suggest that polysaccharides may be one of the active ingredients in aqueous extracts of CW and could be utilized as an effective immune stimulant.

## Figures and Tables

**Table 1:** Effect of HMF on terminal body weight, absolute and relative organ weights of mice.

Group	Initial Body Weight (g)	Terminal Body Weight (g)	Spleen Weight		Thymus Weight	
			Absolute (g)	Relative (%)	Absolute (g)	Relative (%)
Negative control	19.11 ± 0.75 ^a^	20.03 ± 0.65 ^c^	0.103 ± 0.009 ^a^	0.512 ± 0.027 ^ab^	0.047 ± 0.004 ^a^	0.233 ± 0.017 ^a^
CYC	18.82 ± 0.94 ^a^	18.10 ± 0.57 ^a^	0.085 ± 0.005 ^b^	0.471 ± 0.022 ^a^	0.041 ± 0.005 ^a^	0.225 ± 0.023 ^a^
HMF (100 mg/kg)	18.38 ± 0.52 ^a^	18.41 ± 0.53 ^ab^	0.100 ± 0.002 ^a^	0.544 ± 0.014 ^b^	0.042 ± 0.003 ^a^	0.230 ± 0.012 ^b^
HMF (200 mg/kg)	19.11 ± 0.34 ^a^	19.52 ± 0.63 ^bc^	0.108 ± 0.007 ^a^	0.554 ± 0.021 ^b^	0.045 ± 0.003 ^a^	0.232 ± 0.009 ^b^
Positive control	19.32 ± 0.64 ^a^	19.69 ± 0.85 ^c^	0.100 ± 0.008 ^a^	0.510 ± 0.037 ^ab^	0.047 ± 0.005 ^a^	0.238 ± 0.022 ^b^

Values represent Mean ± SD; ^abc^ Means with different superscripts in the same column are significantly different at *p* < 0.05. CYC, cyclophosphamide; CVT, immunostimulatory polysaccharide-rich extract of the root of North American ginseng (*Panax quinquefolius*); HMF, high-molecular-weight fraction of *Cynanchum wilfordii*.

**Table 2:** The chemical and monosaccharide composition of HMFO.

	HMFO
Chemical composition (%)	
Neutral sugar ^1^	63.8 ± 0.9
Uronic acid ^2^	33.1 ± 1.9
KDO ^3^	0.3 ± 0.1
Protein ^4^	2.9 ± 0.2
Component monosaccharide (mg/g)	
Arabinose	86.0 ± 1.6
Galactose	76.5 ± 2.9
Rhamnose	23.0 ± 0.4
Xylose	2.2 ± 0.6
Glucose	241.2 ± 6.5
Mannose	9.0 ± 1.6
Fucose	5.7 ± 0.2
Fructose	ND ^5^
Galacturonic acid	178.8 ± 2.5
Glucuronic acid	4.9 ± 0.2

Values represent Mean ± SD; ^1^ Phenol sulfuric acid method; ^2^ m-Hydroxydiphenyl sulfuric acid method; ^3^ KDO, 2-keto-3-deoxy-d-manno-octulosonic acid; ^4^ Bradford method; ^5^ ND, not detected.

## Supplementary Materials

The following are available online at http://www.mdpi.com/2072-6643/8/10/600/s1. Figure S1: Molecular weight distribution of HMFO by HPGPC.
