# Supplementary material for: Immune-Enhancing Effects of a High Molecular Weight Fraction of Cynanchum wilfordii Hemsley in Macrophages and Immunosuppressed Mice
Source: Nutrients. 2016 Sep 27;8(10):600. doi: 10.3390/nu8100600 (PMC5083988; doi:10.3390/nu8100600)
Supplement: Supplementary file 1 [file nutrients-08-00600-s001.docx]

Supplementary Materials: Immune-Enhancing Effects of a High-Molecular-Weight Fraction from *Cynanchum wilfordii* Hemsley in Macrophages and Immunosuppressed Mice

Mi Jang, Tae-Gyu Lim, Sungeun Ahn, Hee-Do Hong, Young Kyoung Rhee, Kyung-Tack Kim, Eunjung Lee, Jeong Hoon Lee, Yun Ji Lee, Chan Sik Jung, Dae Young Lee and Chang-Won Cho


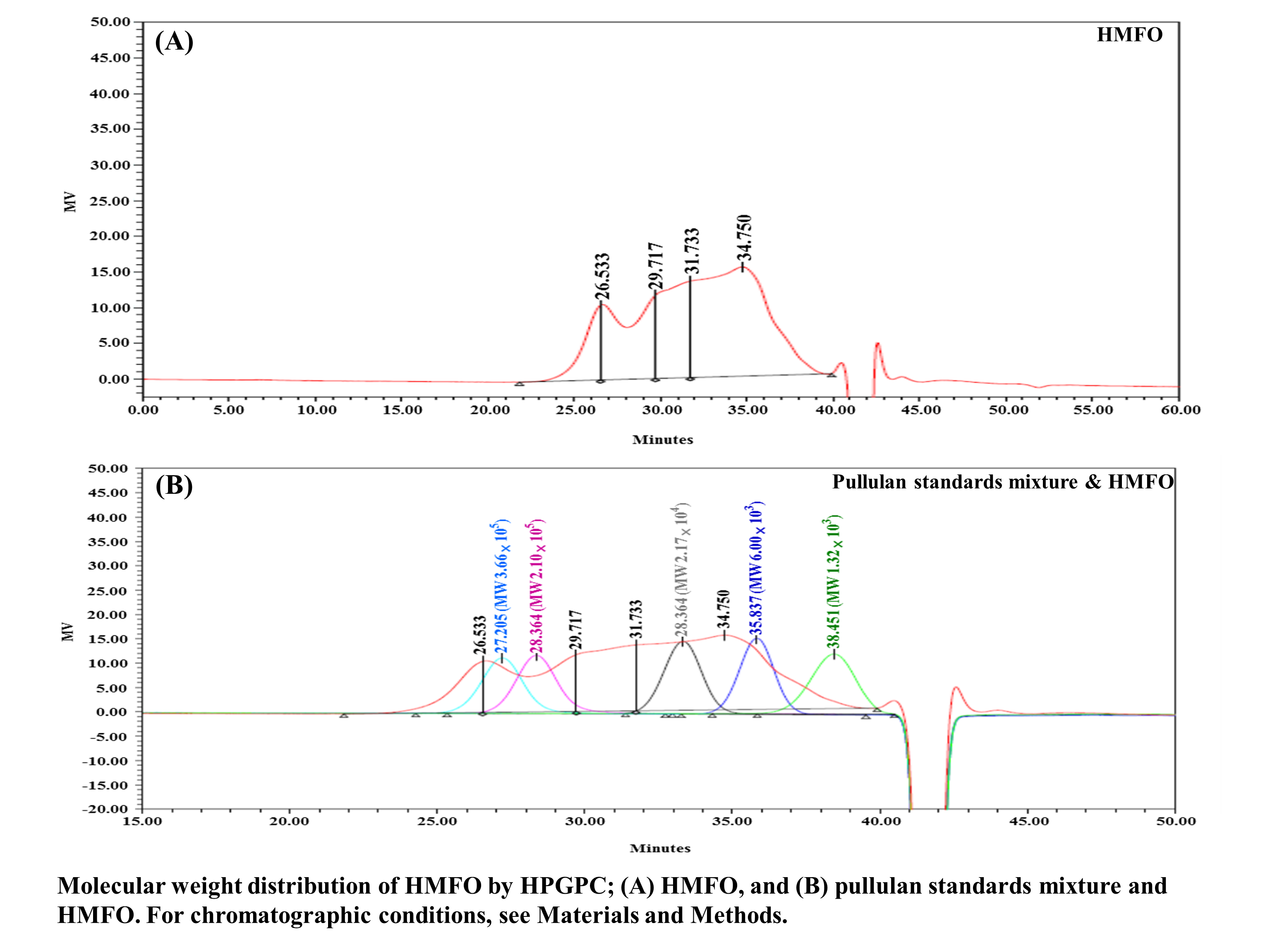


**Figure S1.** Molecular weight distribution of HMFO by HPGPC; (**A**) HMFO, and (**B**) pullulan standards mixture and HMFO. For chromatographic conditions, see Materials and Methods.
